# Influence of pars plana vitrectomy for macular surgery on the medium term intraocular pressure

**DOI:** 10.1371/journal.pone.0241005

**Published:** 2020-10-23

**Authors:** Peer Lauermann, Julia Gebest, Sebastian Pfeiffer, Nicolas Feltgen, Sebastian Bemme, Hans Hoerauf, Christian van Oterendorp

**Affiliations:** 1 Department of Ophthalmology, University Medical Center Goettingen, Goettingen, Germany; 2 Department of Medical Statistics, University Medical Center Goettingen, Goettingen, Germany; University of Warmia, POLAND

## Abstract

**Purpose:**

To evaluate the long-term effect of 20 and 23 gauge pars plana vitrectomy (PPV) on intraocular pressure (IOP).

**Methods:**

Study type: Monocentric retrospective cohort study. 249 eyes of 249 patients undergoing PPV due to epiretinal membrane (EM), idiopathic macular hole (IMH) or vitreoretinal traction (VT) were included. The fellow eye served as control. Exclusion criteria were factors known to influence the IOP, such as cataract surgery during follow-up, extended use of steroids, cryotherapy and silicone oil endotamponade. The relative change of IOP (operated vs. fellow eye) at 6–12 months after surgery was defined as primary endpoint. Secondary endpoints were the relative change of IOP at 3–6 and 12–24 months. Possible influencing cofactors were analysed using ANCOVA.

**Results:**

The primary endpoint did not show a significant IOP reduction of the operated eye relative to the fellow eye (P = 0.089, n = 84). However, the IOP of the operated eye alone was significantly reduced at 6–12 and 12–24 months after surgery (-0.75 ± 2.80 and -1.22 ± 3.29 mmHg, P = 0.008 and 0.007, respectively). The IOP of the fellow eye was also significantly reduced at the 12–24 months period (-0.75 ± 2.73 mmHg, P = 0.008). In the subgroup analysis, sclerotomy size was a significant influencing cofactor, leading to lower IOP after 20G compared to 23G vitrectomy (P = 0.04).

**Conclusion:**

Pars plana vitrectomy did not induce a significant long-term IOP reduction relative to the contralateral eye. However, we observed a IOP lowering potential in 20G vitrectomy.

## Introduction

Following its first description in 1971 by Robert Machemer, vitrectomy via a pars plana approach (PPV) has become a standard surgical procedure in ophthalmology [1]. It is used in various vitreoretinal diseases including macular pathologies, such as idiopathic macular hole (IMH), epiretinal membrane (EM) or vitreomacular traction syndrome (VMT) [2–4]. Different short- and long-term side effects of PPV have been described and extensively studied [5]. Regarding intraocular pressure (IOP) changes, mainly short-term postoperative IOP spikes, which are in part related to gas endotamponades, have been investigated. Additional causes for IOP elevation, such as migration of inflammatory or pigmented cells into the trabecular meshwork (TM), aqueous outflow blockage by neovascularizations or erythrocytes after a bleeding in diabetes, drug-induced effects and postoperative positioning have been discussed [6–12]. Focusing on long-term effects on the IOP after vitrectomy, Stanley Chang was the first to suspect a relationship between glaucoma development and vitrectomy. He suspected that oxidative stress altering the TM could lead to an elevated IOP [13]. Physiologically, there is an oxygen gradient from the retina to the rear of the lens. This gradient is reduced by the vitreous and the lens itself. Consequently, vitrectomy and combined cataract extraction can triple the gradient possibly resulting in increased oxidative stress on the TM [14, 15]. Based on these physiological considerations, several studies observed a significant elevation in long-term IOP after vitrectomy for different pathologies [16–21]. However, other studies failed to show significant changes in IOP [22–25] and some even showed reduced IOP values [26, 27]. In summary, studies focusing on long-term IOP changes after vitrectomy showed controversial results. Additionally, all studies hypothesised IOP **elevations** after surgery with heterogenous study populations. In contrast to these previous studies and based on clinical observations we hypothesised a small long-term IOP reduction, which was investigated in this retrospective cohort study. Inclusion and exclusion criteria were defined such that intra- and postoperative factors influencing mid- and long term IOP were largely avoided.

## Materials and methods

### Study population

After approval from the local ethics committee (Ethik-Kommision der Universitätsmedizin Göttingen, Von-Siebold-Straße 3, 37075 Göttingen), 596 patients of the Department of Ophthalmology at the University Medical Center Göttingen with prior PPV were screened retrospectively for inclusion in the study. Interventions were carried out by 4 different surgeons. Inclusion criteria were ppv for idiopathic macular hole (IMH), epiretinal membrane (EM) or vitreomacular traction (VMT). Exclusion criteria (for study and fellow eyes) included a follow-up duration of less than 3 months, age under 18 years, the extended use of steroid eye drops (i.e. exceeding our standard procedure for postoperative care in our clinic (Dexagentamycin eye drops 5 times daily for one week, followed by reduction by one drop per week)), surgical procedures known to influence the IOP (intraoperative retinal cryotherapy or laser-photocoagulation, combined cataract surgery or cataract surgery during follow-up period, previous ppv and use of silicon oil endotamponade) and IOP-influencing diseases (current or history of retinal detachment, known steroid response, history of glaucoma, uveitis, severe eye trauma, diabetic retinopathy, diabetic macular edema and vitreous hemorrhage). All IOP measurements were carried out with Goldmann applanation tonometry.

### Surgical procedure

For 20G sclerotomies conjunctiva was opened in the temporal sector over about 3–4 clock hours and additionally over 1 clockhour within the nasal upper quadrant. In contrast, 23 G trocars have been placed transconjunctivally. For 20G vitrectomy, sclerotomies were always sutured. If a gas endotamponade was necessary, a 100% filling was aimed for.

### Follow-up time intervalls, study endpoints and additional influencing variables

IOP at different time-points before and after vitrectomy was measured for the study eye and the fellow eye. For statistical evaluation of the time course, the examination time-points were grouped into **defined time intervals**:

- up to 6 days before surgery (**baseline**)
- up to 6 days after surgery (**postoperative**)
- from inpatient discharge up to 3 months after surgery (**0-3m**)
- 3 months post-surgery to < 6 months post-surgery (**3-6m**)
- 6 months post-surgery to < 12 months post-surgery (**6-12m**)
- 12 months post-surgery to < 24 months post-surgery (**12-24m**)
- ≥ 24 months post-surgery (≥**24m**)

If multiple IOP data was available for a given time interval the mean IOP was used for the statistical analysis.

From the IOP data of the study eyes and their respective (contralateral) control eyes two parameters were calculated consecutively:

1. The IOP change (IOPc) for each eye, which was defined as the difference between the baseline IOP, measured preoperatively, and the follow-up IOP at one of the above defined time intervals:
  *IOP*_*c*_ *= IOP(follow-up)–IOP(baseline)*
  (IOPc becomes negative when the IOP drops postoperatively.)
2. To correct for systematic errors the IOP change of the study eye was set into relation to the IOP change of the contralateral control eye. The resulting difference was termed ΔIOPc.
  ΔIOP_c_ = IOP_c_(control eye)–IOP_c_(study eye)
  (If the IOPc of the study eye exceeds the IOPc of the control eye the ΔIOPc-value becomes positive.)

The **primary endpoint** was defined as ΔIOPc at the 6–12 month time interval. The null hypothesis was defined as *H0*: *ΔIOP*_*c*_ *= 0* and *H1*: *ΔIOP*_*c*_ *> 0*.

As **secondary endpoints** we defined the ΔIOPc at 3–6 months and ΔIOPc at 12–24 months.

For the study eye, the influence of lens status, vitrectomy, endotamponade, sex and the covariates, IOPbaseline, age, spherical equivalent and number of IOP lowering eyedrops on the primary endpoint was calculated using ANCOVA. In another analysis for both eyes, the influence of surgery (yes/no), lens status, sex and the covariates IOP(baseline), age, spherical equivalent and number of IOP lowering eyedrops was calculated.

### Statistical methods

Statistical analysis was done using SAS Visual Statistics (SAS Institute Inc., Cary, USA) and Prism 6 (6.07, Graph Pad, La Jolla, USA). The t-test and ANOVA were used to determine statistical differences in the mean values of groups. Analysis of Covariance (ANCOVA) was used to investigate the influence of selected parameters on the results. The data was presented either as mean ± standard error of the mean for normally distributed data or median / interquartile range for not normally distributed data. P < 0,05 were considered significant.

## Results

### Population characteristics and IOP time course

A total of 249 patients (137 women [55%], 122 men [45%]) were included in the study. Details of the study cohort are reported in Table 1. Of the 249 patients, the majority was phakic in the study eye (231 patients (92,8%)). If postoperative cataract extraction was performed the study observation was ended at this timepoint.

**Table 1:** Study population.

baseline characteristics	n = 249
**sex**	women: n = 137 (55%)
	men: n = 122 (45%)
**age:** mean ± SD [years]	70 ± 6.3 (range 44–100).
**study eye side**	left: n = 124 (49.8%)
	right: n = 125 (50.2%)
**diagnosis**	EM: n = 140 (56.2%)
	IMH: n = 100 (40.2%)
	VMT: n = 5 (2%)
	IMH + EM: n = 4 (1.6%)
**mean spherical equivalent** [dpt]	study eyes: +0.35 ± 2,59
	fellow eyes: +0.45 ± 2,63
**lens status:** phakic	n = 231 (92.8%)
**IOP**_**baseline**_: mean ± SD [mmHg]	study eyes: 15.67 ± 2.80 (range 9–24)
	fellow eyes: 15.49 ± 2.90 (range 6–23)

Sclerotomy size was 20G in 202 cases (81.1%) and 23G in 47 cases (18.9%). Gas endotamponade with C2F6 was used in 39 (15.7%), SF6 in 73 (29.3%) and air in 19 (7.6%) patients. In 118 (47.4%) cases, no endotamponade was used. The peeling dye Brilliant Peel was used in 103 (41.4%) cases. In 56 (22.5%) cases, no dye was used. Other dyes were used less frequently: Brilliant Blue n = 27 (10.8%), Indocyanin Green n = 23 (9.2%), ILM-Blue n = 22 (8.8%), Dual Blue: n = 16 (6.4%), Methylen Blue n = 1 (0.4%), Trypan Blue n = 1 (0.4%).

In the dependent t-test analysis for IOP differences between IOP(baseline) and IOP at each interval, the mean IOP of the study eyes showed a significant rise postoperatively (IOPc = 1.93mmHg, P < 0.001). In the long-term course, both, study eyes’ and fellow eyes’ mean IOP showed significant reductions. The IOP of the study eyes was significantly reduced after 6–12 months (IOPc = -0.75 mmHg, P = 0.008) and 12–24 months (IOPc = -1.22mmHg, P = 0.007). The IOP of the fellow eyes was significantly reduced after inpatient discharge (IOPc = -0.55 mmHg, p < 0.001) and after 12–24 month(IOPc = -0,75 mmHg, P = 0.008) (Table 2).

**Table 2:** IOP time course and statistics of study eyes and fellow eyes.

Visits	study eye (S)	N	IOP [mmHg]					
	fellow eye (F)		Mean	SD	SEM	Min	Median	Max
**preoperative**	S*	249	15.67	2.81	0.18	9	16	24
	F	249	15.49	2.9	0.18	6	15	23
postoperative*	S*	225	17.6	5.42	0.36	2	17	39
	F							
discharge^**^	S	244	15.88	4.56	0.29	4	16	33
	F^**^	232	14.94	3.05	0.37	6	15	23
**0-3m**	S	184	15.68	3.70	0.27	6	16	28
	F	104	14.46	2.76	0.27	9	14	24
**3-6m**	S	91	14.68	3.63	0.38	5	14	30
	F	65	14.67	3.04	0.37	8	14.5	23
6-12m*	S*	98	14.91	2.80	0.28	10	14.5	22
	F	84	15.25	2.49	0.27	10	15.25	20
12-24m*^,^ ^**^	S*	51	14.72	3.29	0.46	7.5	15	24
	F^**^	50	14.74	2.73	0.39	6	15	20.5
**>24m**	S	18	14.93	2.49	0.59	10.67	15	20
	F	18	15.15	2.4	0.57	10.57	15	21

At the postoperative timepoint only the study eye IOP was available for most datasets

*paired t-tests for study eyes: postoperative vs preoperative: +1.91 mmHg, P < 0.001; 6-12m vs preoperative: -0.75 mmHg, P = 0.008; 12-24m vs preoperative: -1.22mmHg, P = 0.007

**paired t-tests for fellow eyes: inpatient discharge vs preoperative: -0.55mmHg, P < 0.001; 12-24m vs preoperative: -0.75, P = 0.008.

### Endpoint analysis

**Fig 1:**
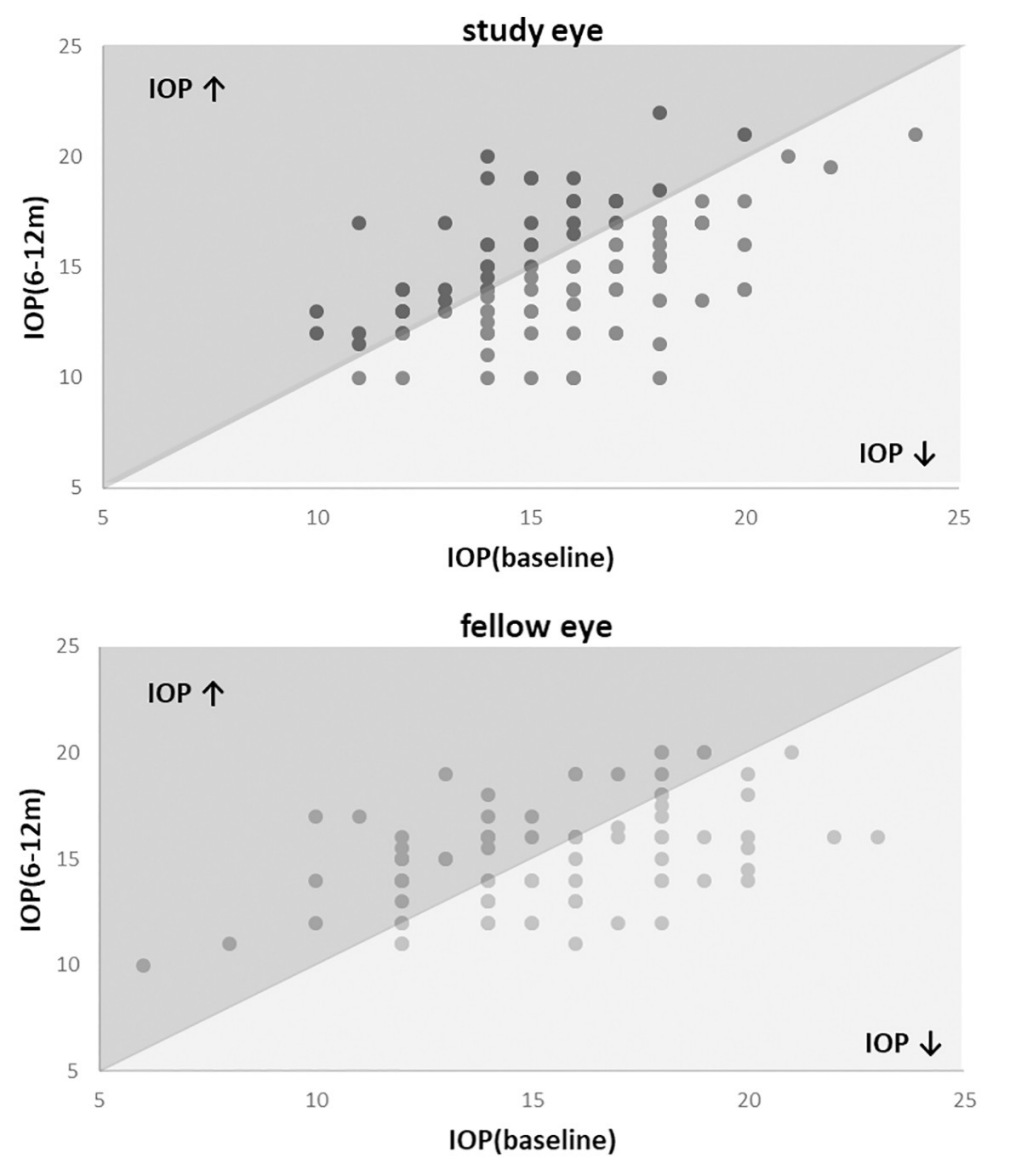
**IOP change scatter plot of study (top) and fellow eyes (bottom).** Data in mmHg. Data points in the darker area indicate an IOP decrease after surgery.

For endpoint analyses, only patients with IOP data for *both eyes* were included. For this reason the single eye data presented in the endpoint analysis may differ from the data in Table 2, where all available datasets were shown. Six to twelve months after surgery, the primary endpoint, the IOP of the study eyes was -0.66 ± 2.78 mmHg (mean ± SD of IOPc) lower than at baseline (n = 84, P = 0.008). The IOP change of the fellow eyes was smaller (-0.17 ± 2.98 mmHg, mean ± SD) and not statistically significant (P = 0.61). The ΔIOPc (difference between IOP change of study and fellow eye) showed a trend towards a larger IOP reduction in the study eyes (P = 0.089) but the difference was not statistically significant (Table 3, Fig 1).

**Table 3:** IOP change at the primary endpoint.

	n	mean	SD	Min	Max	P
**IOP**_**c**_ **[mmHg] of study eyes**	84	-0.66	2.78	-8.00	6.00	*0*.008^*^
**IOP**_**c**_ **[mmHg] of fellow eyes**	84	-0.17	2.98	-7.00	7.00	0.61
**ΔIOP**_**c**_ **[mmHg]**	84	0.49	0.29	-7.00	8.50	0.0893

There was a significant IOP reduction in the study eye, but the relative reduction considering the IOP change of the fellow eye was not significantly higher.

**Fig 2:**
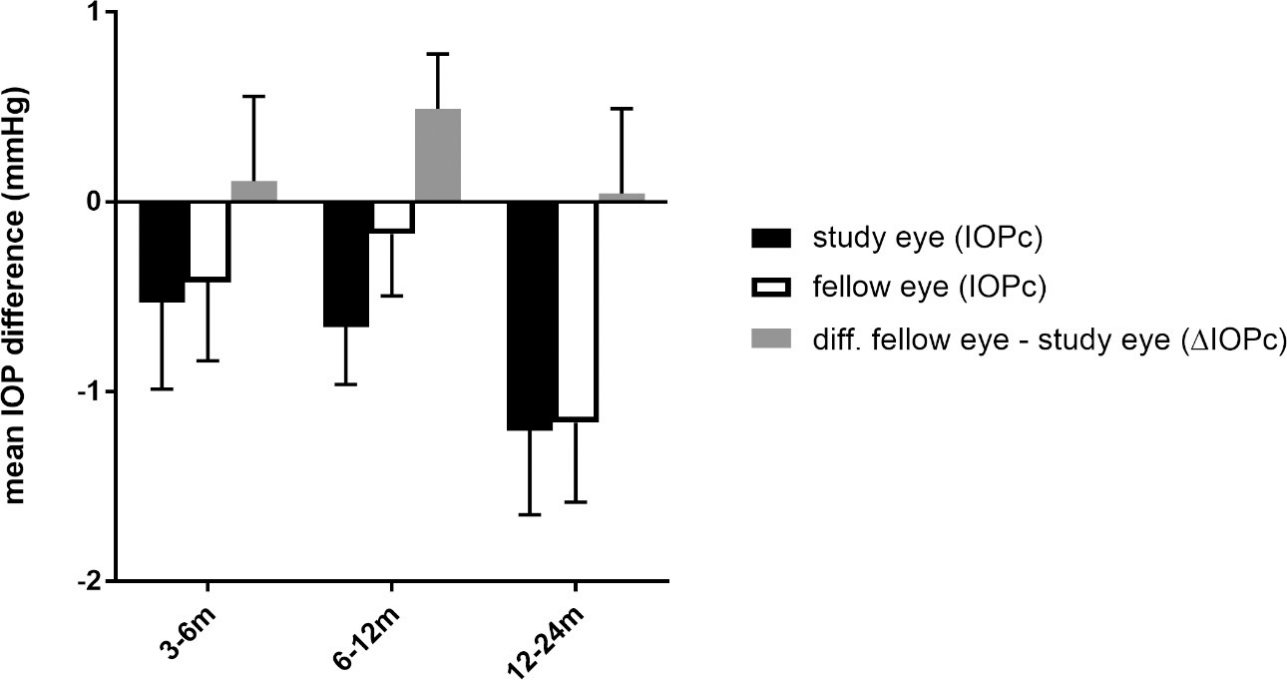
Mean IOP difference at primary and secondary endpoints, bars indicating standard error of the mean (SEM), P(3-6m) = 0.81, P(6-12m) = 0.09, P(12-24m) = 0.92. Note that a positive fellow—study eye difference indicates a more pronounced change of IOP of the study eye relative to the fellow eye.

Regarding secondary endpoints, both, the change of IOP of the study eye at 3–6 and 12–24 months were negative (mean ± SD of IOPc: -0.53 ± 3.68 mmHg and -1.21 ± 3.13 mmHg, respectively), indicating a postoperative IOP reduction. However, the relative change of IOP (ΔIOPc) was not significantly different from 0 at both time periods (P = 0.81 and P P = 0.92) (Tables 4 and 5, Fig 2).

**Table 4:** IOP change at secondary endpoint 3–6 months.

eye	n	mean	SD	Min	Max	P
**IOP**_**c**_ **[mmHg] of study eyes**	65	-0.53	3.68	-7.00	15.00	
**IOP**_**c**_ **[mmHg] of fellow eyes**	65	-0.42	3.34	-10.00	8.00	
**ΔIOPc [mmHg]**		-0.11	3.61	-16.00	9.00	0.81

The relative change of IOP (ΔIOPc) was not significantly different from zero.

**Table 5:** IOP change at secondary endpoint 12–24 months.

IOPc / eye	n	mean	SD	Min	Max	P
**IOP**_**c**_ **[mmHg] of study eyes**	50	-1.21	3.13	-7.00	10.00	
**IOP**_**c**_ **[mmHg] of fellow eyes**	50	-1.16	2.96	-11.00	7.00	
**ΔIOPc [mmHg]**		-0.04	3.16	-9.00	11.00	0.92

The relative change of IOP (ΔIOPc) was not significantly different from zero.

### Additional influencing variables (ANCOVA analysis)

The patients included in the study showed different patient characteristics (lens status, sex, age, spherical equivalent) and different surgical characteristics such as sclerotomy size or endotamponade. Using ANCOVA the influence of these different characteristics on the IOP change after 6–12 month was analyzed (Table 6). For all possible influencing variables, only the sclerotomy size showed a significant effect on the IOPc.

**Table 6:** ANCOVA of influencing variables.

variable	parameter	n	F	P
**lens status**	phakic	87	0.28	0.6
	pseudophakic			
**sclerotomy size**	20G	87	4.19	0.043*
	23G			
**endotamponade**	C2F6	87	0.80	0.498
	air			
	SF6			
	none			
**sex**	male	87	0.67	0.414
	female			
**age**	in years	87	0.02	0.877
**spherical equivalent**	value of spherical equivalent	87	0.00	0.977
**number of IOP lowering eyedrops**	N	87	0.06	0.815
**surgery (yes/no)**	yes	174 (both eyes)	1.68	0.196
	no			

**Fig 3:**
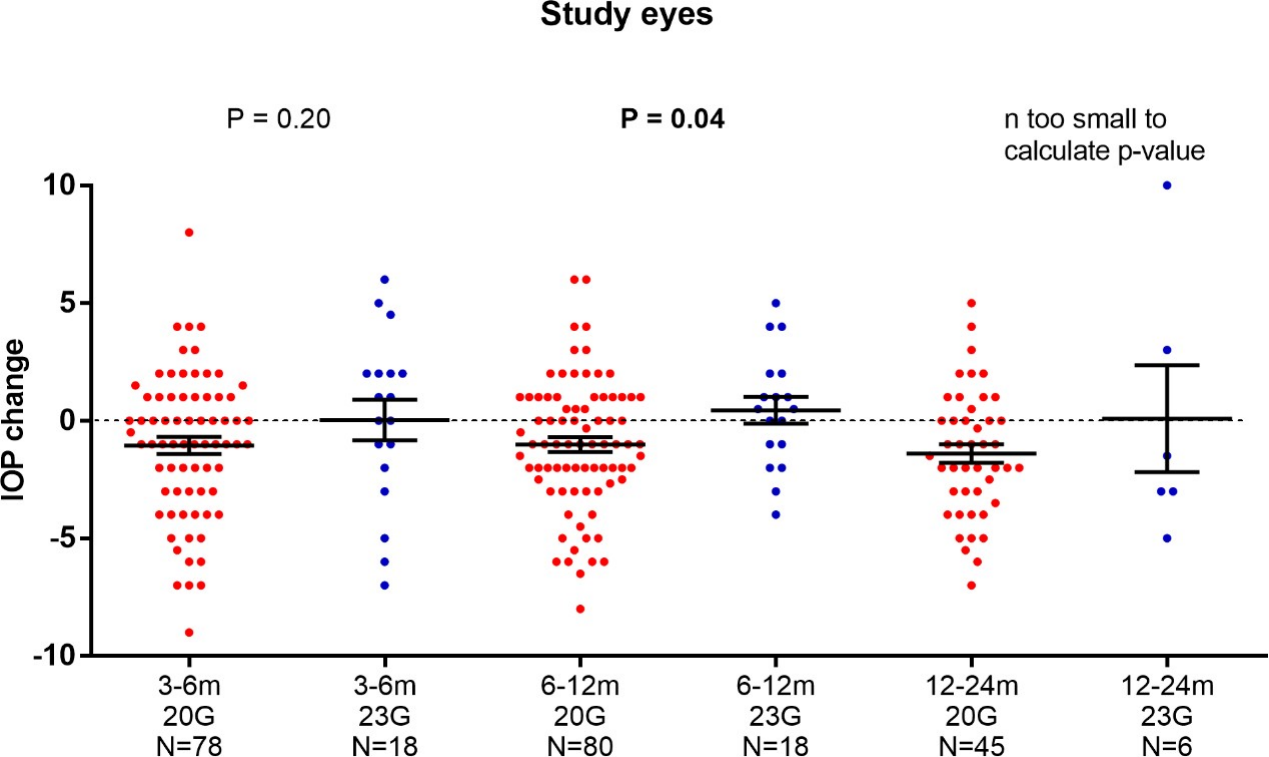
IOPc of 20G versus 23G vitrectomized study eyes. At the primary endpoint (6-12m) the difference was significantly different (P = 0.04) using unpaired t-test (Error bars indicating mean ± SEM).

**Fig 4:**
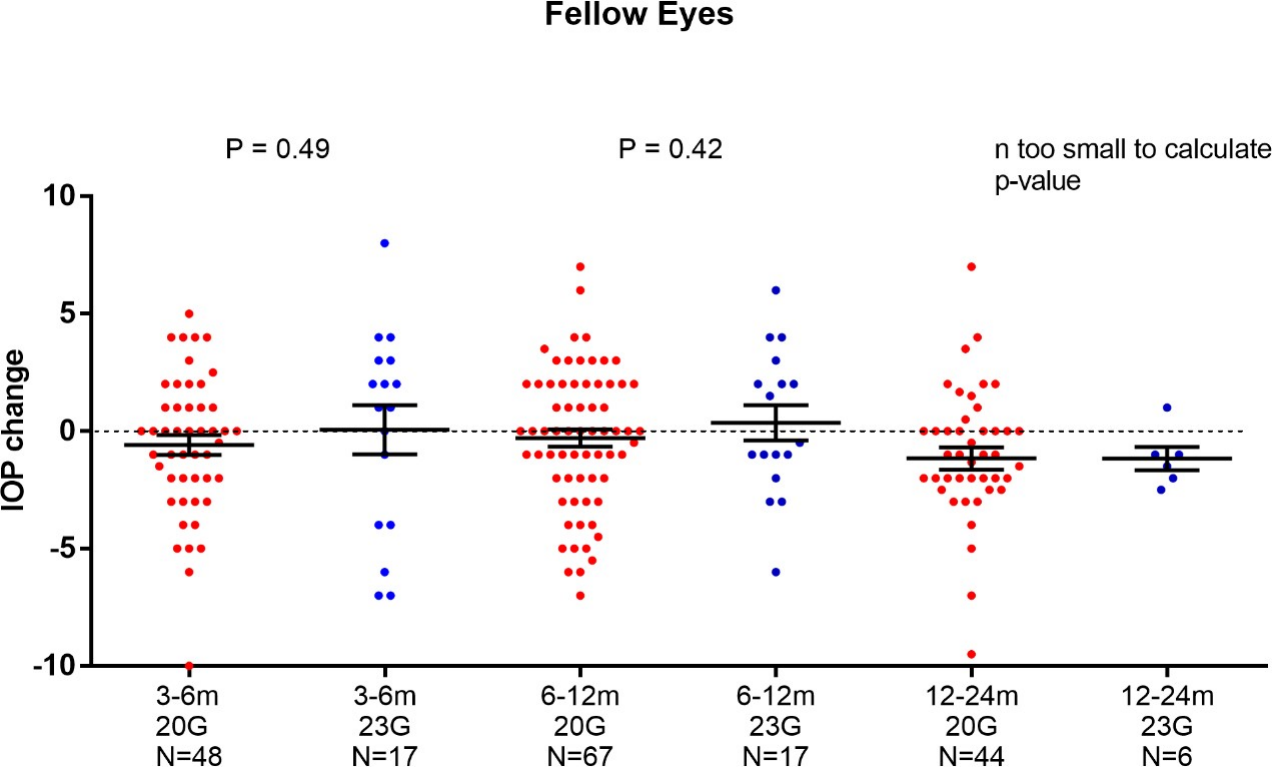
IOP_c_ of 20G versus 23G in fellow eyes. There was no significant difference between both groups using unpaired t-test.

As the sclerotomy size was a significant influencing factor on IOPc in the ANCOVA analysis, a post-hoc analysis was performed. The change of IOP in the study eyes was significantly different between 20G and 23G at the 6–12 months period, with lower IOP values for the 20G group (mean ± SD of IOPc: -1.02 ± 0.31 vs 0.44 ± 0.52 mmHg; P = 0.04, Fig 3). No significant difference was seen in the fellow eyes (mean ± SD of IOPc: -0.29 ± 0.36 vs 0.35 ± 0.74 mmHg; P = 0.42, Fig 4). The relative change of IOP for the 20G vitrectomized eyes showed a strong trend towards a significantly larger IOP decrease (mean ± SD of ΔIOPc: -0.59 ± 0.31 mmHg; P = 0.05). For the 23G vitrectomy subgroup the difference of IOPc between both eyes was not significant (mean ± SD of ΔIOPc: 0.12 ± 0.74 mmHg; P = 0.87).

In the ANCOVA the use of different endotamponades was not a significant influencing factor on the IOP change. However, IOP was reduced at every observation period when no endotamponade was used. Additionally, most surgical procedures without the use of any endotamponade were done using the 20G technique, which itself was a significant influencing factor on the IOPc. Therefore, this analysis might have been significantly biased.

## Discussion

Previously published studies investigating the effect of PPV on medium to long term IOP hypothesized a long term IOP increase after PPV. Several authors assumed that an increase of oxidative stress, due to the elimination of the protective influence of the vitreous, and, after combined surgery, also of the lens, may lead to an increased outflow resistance in the TM, resulting in an IOP increase after PPV [15–21]. However, the results of these previous studies were mostly contradicting this hypothesis [25, 26], some studies in fact indicated an IOP lowering effect in the long term [25–27]. Significant differences in the design and inclusion/exclusion criteria of these previous studies hinder valid conclusions. We therefore decided to conduct a study, which primarily hypothesized an IOP lowering effect and applied stringent selection criteria to control for possible confounding factors on the long term IOP course.

In our study, we observed a small but significant IOP decrease in the study eye. However, also the IOP of the fellow eyes slightly decreased, even reaching statistical significance for the 12–24 months follow-up group (P = 0.008). A similar phenomenon was observed in the study of Mi and Thompson [25], but the authors, like us, cannot present a reasonable explanation for this.

For the whole study population the difference between study and fellow eye IOP reduction never reached statistical significance. However, in the subgroup of eyes operated with 20G vitrectomy, the trend was stronger (P = 0.05). The ANCOVA identified sclerotomy size (20/23G) as a significant influencing factor on ΔIOPc (P = 0.043) and in the post hoc analysis comparing the IOPc of the study eyes with 20G versus 23G vitrectomy the difference was also significant (P = 0.04). Thus, we conclude that the IOP lowering effect of pars plana vitrectomy was mainly connected with 20G, but not 23G (and presumably smaller gauge) vitrectomy. A possible explanation might be the higher stability of the more rigid 20G vitrectome [28] possibly leading to a more effective removal of the vitreous in the outer periphery of the retina, which could potentially facilitate uveoscleral outflow of the aqueous. However, this theory has not yet been proven in studies. One fundamental difference between PPV techniques is the necessity to open the conjunctiva in 20G PPV compared to 23G. Altered perfusion of episcleral veins in consequence of healing conjunctival and tenonal tissue in 20G PPV may contribute to postoperative IOP changes.

IOP differences depending on the sclerotomy size have already been investigated in several studies. For the 23G vitrectomy, mainly short-term IOP-lowering effects were seen [29–32]. For 20G vitrectomy, however, no IOP lowering effect was seen [10]. The differences were explained by a slight leakage through the seamless 23G sclerotomies. Regarding long term effects, other studies found no difference between 23 and 20G vitrectomies at all [32, 33]. However, the comparability with our study is limited due to different inclusion criteria. In the studies mentioned above, patients with retinal detachment and/ or oil endotamponade [32] as well as combined IOL implantation [33] were included. In our study, these treatments were defined as exclusion criteria due to their possible influence on the IOP. In addition to the sclerotomy size the influence of different endotamponades was analyzed. A lower IOP was seen in patients without any use of endotamponades. However, these were operated almost entirely with 20G vitrectomy (95%), which renders this finding rather unreliable.

Significant limitations of this study come with its restrospective design. Among these are a possible selection bias during follow up, unequal group sizes for co-variates, such as sclerotomy size and use of endotamponades, mainly single IOP measurements at the various timepoints, the lack of information about the angle width, central corneal thickness and axial length–all factors potentially incfluencing IOP, and decreasing group sizes for the late follow-up timepoint 12–24 months. As more than 92% of patients were phacic at the time of PPV, the possible development of cataracta complicate and a resulting IOP increase due to the thickening of the lens could have influenced the follow up data. On the other hand patients were excluded at the timepoint of phacoemulsification, possibly excluding higher IOPs of the later time course.

Therefore a prospective study would be desirable to better control for confounding factors. A main obstacle for such a study, however, would be the current widespread replacement of 20G vitrectomy for routine macula surgery by smaller gauge approaches.

Using the mean of multiple IOP values for different timepoints could have altered the results. We have chosen to calculate the mean because we consider IOP values in general normally distributed. Using the median would have reduced the influence of single outliers relative to the majority of the other IOP values. However, as the number of values from which the mean was calculated was usually small, mainly 2 to 3 values, the difference between mean and median would have been very small.

The potentially IOP-lowering effect of 20G vitrectomy is an interesting finding but its effect size is too small to consider it a therapeutic option for glaucoma patients. The potential advantages of smaller gauge vitrectomy by far exceed the potential positive effect of 20G vitrectomy on IOP.

## Supporting information

S1 Data (RTF)

S2 Data (RTF)

S3 Data (RTF)

S4 Data (XLSX)
