# Supplementary material for: Influence of pars plana vitrectomy for macular surgery on the medium term intraocular pressure
Source: PLoS One. 2020 Oct 23;15(10):e0241005. doi: 10.1371/journal.pone.0241005 (PMC7584206; doi:10.1371/journal.pone.0241005)
Supplement: S1 Data — (RTF) [file pone.0241005.s001.rtf]

Baseline-Characteristics	
Table 1.1: age and sex at surgery	

Variable	sex	N	NMiss	Mean	SEM	STD	MIN	Q1	Median	Q3	MAX	Method	t-Test:
t-Value	t-Test:
DF	t-Test:
p-Value	Difference:
Mean	Difference:
LCL	Difference:
UCL	
age	female	137	0	70.07	0.56	6.56	54.05	65.66	69.94	73.56	100.70	Pooled	0.08	247	0.9343	0.07	-1.51	1.64	
	male	112	0	70.01	0.56	5.91	44.59	67.49	70.13	73.62	83.97		.	.	.	.	.	.	

Parameters: alpha=5%, H0=0	


N	NMiss	Mean	STD	SEM	Min	Q1	Median	Q3	Max	Mean (95%LCL)	Mean (95%UCL)	
249	0	70.04	6.26	0.40	44.59	66.84	70.05	73.62	100.70	69.26	70.82	

Table 1.2: use of systemic steroids	


	systemic steroids		
sex	inhalative (i)	intranasal (i.n.)	oral (p.o.)	none (k)	Total	
female	5 (3.6%)	1 (0.7%)	4 (2.9%)	127 (92.7%)	137 (55%)	
male	2 (1.8%)	0 (0%)	1 (0.9%)	109 (97.3%)	112 (45%)	
sum	7 (2.8%)	1 (0.4%)	5 (2%)	236 (94.8%)	249 (100%)	


Table 1.3: study eye	


	study eye		
sex	left	right	total	
female	73 (53.3%)	64 (46.7%)	137 (55%)	
male	51 (45.5%)	61 (54.5%)	112 (45%)	
total	124 (49.8%)	125 (50.2%)	249 (100%)	

baseline characteristics	
table 2.1: IOP - Baseline	


Variable	surgery	N	NMiss	Mean	SEM	STD	MIN	Q1	Median	Q3	MAX	Method	t-Test:
t-Value	t-Test:
DF	t-Test:
p-Value	Difference:
Mean	Difference:
LCL	Difference:
UCL	
IOP_Mean_bl	yes	249	0	15.67	0.18	2.81	9.00	14.00	16.00	17.00	24.00	Pooled	0.69	496	0.4903	0.18	-0.33	0.68	
	no	249	0	15.49	0.18	2.90	6.00	14.00	15.00	18.00	23.00		.	.	.	.	.	.	

Parameters: alpha=5%, H0=0	
table 2.2: sphere+ 1/2 cylinder 	


Variable	surgery	N	NMiss	Mean	SEM	STD	MIN	Q1	Median	Q3	MAX	Method	t-Test:
t-Value	t-Test:
DF	t-Test:
p-Value	Difference:
Mean	Difference:
LCL	Difference:
UCL	
Sph_q	yes	249	0	0.35	0.16	2.60	-15.00	-0.50	0.75	2.00	7.00	Pooled	-0.44	496	0.6607	-0.10	-0.56	0.36	
	no	249	0	0.45	0.17	2.63	-12.00	-0.25	1.00	2.00	7.50		.	.	.	.	.	.	

Parameters: alpha=5%, H0=0	

table 2.3: diagnosis	


	Diagnosis		
Study eye	Epiretinal gliosis (EG)	Idiopathic macular hole (MF)	Vitreomacular traction (VRT)	MF+EG	VMT
Vitreomacular traction syndrome	keine (k)	Total	
yes	140 (56.2%)	100 (40.2%)	3 (1.2%)	4 (1.6%)	2 (0.8%)	0 (0%)	249	
no	0 (0%)	0 (0%)	0 (0%)	0 (0%)	0 (0%)	249 (100%)	249	
Total	140	100	3	4	2	249	498	

Table 2.4: Vitrectomy	


	Vitrectomy		
Study eye	20G	23G	30G	k	Total	
yes	201 (80.7%)	47 (18.9%)	1 (0.4%)	0 (0%)	249	
no	0 (0%)	0 (0%)	0 (0%)	249 (100%)	249	
Total	201	47	1	249	498	

Table 2.5: Endotamponade	


	Endotamponade		
Study eye	C2F5	C2F6	Air	SF6	k	Total	
yes	1 (0.4%)	38 (15.3%)	19 (7.6%)	73 (29.3%)	118 (47.4%)	249	
no	0 (0%)	0 (0%)	0 (0%)	0 (0%)	249 (100%)	249	
Total	1	38	19	73	367	498	

Table 2.6: Peeling due	


	Peeling due		
Study eye	Brilliant Blau (BB)	Brilliant Peel (BP)	Dual Blue (DB)	Methylenblau (MB)	Indocyaningrün (ICG)	Trypanblau (TB)	ILM-Blue	keine (k)	Total	
yes	27 (10.8%)	103 (41.4%)	16 (6.4%)	1 (0.4%)	23 (9.2%)	1 (0.4%)	22 (8.8%)	56 (22.5%)	249	
no	0 (0%)	0 (0%)	0 (0%)	0 (0%)	0 (0%)	0 (0%)	0 (0%)	249 (100%)	249	
Total	27	103	16	1	23	1	22	305	498	


Lense state	


Table 2.7-1: Contingency table	


	Lense state		
Study eye	phak	pseudophak	Total	
yes	231 (49.9%;92.8%)	18 (51.4%;7.2%)	249 (50%)	
no	232 (50.1%;93.2%)	17 (48.6%;6.8%)	249 (50%)	
Total	463 (93%)	35 (7%)	498 (100%)	

Table 2.7-2: Confidence intervals, Difference of rates, Chi square test, Fisher's exact test	


	Confidence intervals		
	Asymptotic	Exact	Chi square test		
Study eye	Rate	95% LCL	95% UCL	95% LCL	95% UCL	DF	Test-
statistic	p-value	Fisher's
exact test	
yes	0.9277	.	.	0.8882	0.9566	1	0.0307	0.8608	1.0000	
no	0.9317	.	.	0.8929	0.9597	.	.	.	.	
Total	0.9297	.	.	0.9036	0.9506	.	.	.	.	
Difference	-0.0040	-0.0489	0.0409	.	.	.	.	.	.	


Table 3.1: IOP (Intraocular pressure)	


Visit	Study eye	N	NMiss	Mean	STD	SEM	Min	Q1	Median	Q3	Max	Mean (95%LCL)	Mean (95%UCL)	
Pre surgery	yes	249	0	15.67	2.81	0.18	9.00	14.00	16.00	17.00	24.00	15.32	16.02	
	no	249	0	15.49	2.90	0.18	6.00	14.00	15.00	18.00	23.00	15.13	15.86	
	Total	498	0	15.58	2.85	0.13	6.00	14.00	16.00	18.00	24.00	15.33	15.83	
Post OP	yes	225	0	17.60	5.42	0.36	2.00	14.00	17.00	20.00	39.00	16.89	18.32	
	no	1	0	13.00	.	.	13.00	13.00	13.00	13.00	13.00	.	.	
	Total	226	0	17.58	5.41	0.36	2.00	14.00	17.00	20.00	39.00	16.87	18.29	
Discharge	yes	244	0	15.88	4.56	0.29	4.00	13.00	16.00	18.00	33.00	15.30	16.45	
	no	232	0	14.94	3.05	0.20	6.00	13.00	15.00	17.00	23.00	14.55	15.34	
	Total	476	0	15.42	3.92	0.18	4.00	13.00	15.00	18.00	33.00	15.07	15.78	
Entl<Monat<3	yes	180	0	15.71	3.73	0.28	6.00	14.00	16.00	18.00	28.00	15.16	16.26	
	no	101	0	14.45	2.74	0.27	9.00	12.00	14.00	16.00	24.00	13.90	14.99	
	Total	281	0	15.25	3.46	0.21	6.00	13.00	15.00	17.00	28.00	14.85	15.66	
3<=Monat<=6	yes	95	0	14.70	3.56	0.37	5.00	13.00	14.00	16.00	30.00	13.97	15.43	
	no	67	0	14.74	3.04	0.37	8.00	12.50	14.50	16.00	23.00	14.00	15.48	
	Total	162	0	14.72	3.35	0.26	5.00	13.00	14.50	16.00	30.00	14.20	15.24	
6<Monat<=12	yes	98	0	14.91	2.80	0.28	10.00	13.00	14.50	17.00	22.00	14.35	15.48	
	no	84	0	15.25	2.49	0.27	10.00	14.00	15.25	17.00	20.00	14.71	15.79	
	Total	182	0	15.07	2.66	0.20	10.00	13.00	15.00	17.00	22.00	14.68	15.46	
12<Monat<=24	yes	51	0	14.72	3.29	0.46	7.50	12.00	15.00	17.67	24.00	13.79	15.64	
	no	50	0	14.74	2.73	0.39	6.00	13.00	15.00	16.00	20.50	13.96	15.51	
	Total	101	0	14.73	3.01	0.30	6.00	13.00	15.00	17.00	24.00	14.13	15.32	
24<Monat	yes	18	0	14.93	2.49	0.59	10.67	13.00	15.00	16.00	20.00	13.69	16.16	
	no	18	0	15.15	2.40	0.57	10.67	14.00	15.00	17.00	21.00	13.96	16.34	
	Total	36	0	15.04	2.41	0.40	10.67	13.50	15.00	16.50	21.00	14.22	15.85	


Table 3.2: Number of steroid eye drops (only Study eye)	


Visit	Study eye	N	NMiss	Mean	STD	SEM	Min	Q1	Median	Q3	Max	Mean (95%LCL)	Mean (95%UCL)	
Pre surgery	yes	249	0	0.00	0.00	0.00	0.00	0.00	0.00	0.00	0.00	.	.	
	no	249	0	0.00	0.00	0.00	0.00	0.00	0.00	0.00	0.00	.	.	
	Total	498	0	0.00	0.00	0.00	0.00	0.00	0.00	0.00	0.00	.	.	
Post OP	yes	225	0	1.00	0.09	0.01	0.00	1.00	1.00	1.00	2.00	0.99	1.01	
	no	1	0	0.00	.	.	0.00	0.00	0.00	0.00	0.00	.	.	
	Total	226	0	1.00	0.12	0.01	0.00	1.00	1.00	1.00	2.00	0.98	1.01	
Discharge	yes	244	0	0.99	0.09	0.01	0.00	1.00	1.00	1.00	1.00	0.98	1.00	
	no	232	0	0.00	0.00	0.00	0.00	0.00	0.00	0.00	0.00	.	.	
	Total	476	0	0.51	0.50	0.02	0.00	0.00	1.00	1.00	1.00	0.46	0.55	
Entl<Monat<3	yes	180	0	0.18	0.42	0.03	0.00	0.00	0.00	0.00	2.00	0.12	0.24	
	no	101	0	0.00	0.00	0.00	0.00	0.00	0.00	0.00	0.00	.	.	
	Total	281	0	0.11	0.35	0.02	0.00	0.00	0.00	0.00	2.00	0.07	0.16	
3<=Monat<=6	yes	95	0	0.01	0.10	0.01	0.00	0.00	0.00	0.00	1.00	-0.01	0.03	
	no	67	0	0.00	0.00	0.00	0.00	0.00	0.00	0.00	0.00	.	.	
	Total	162	0	0.01	0.08	0.01	0.00	0.00	0.00	0.00	1.00	-0.01	0.02	
6<Monat<=12	yes	98	0	0.00	0.00	0.00	0.00	0.00	0.00	0.00	0.00	.	.	
	no	84	0	0.00	0.00	0.00	0.00	0.00	0.00	0.00	0.00	.	.	
	Total	182	0	0.00	0.00	0.00	0.00	0.00	0.00	0.00	0.00	.	.	
12<Monat<=24	yes	51	0	0.00	0.00	0.00	0.00	0.00	0.00	0.00	0.00	.	.	
	no	50	0	0.00	0.00	0.00	0.00	0.00	0.00	0.00	0.00	.	.	
	Total	101	0	0.00	0.00	0.00	0.00	0.00	0.00	0.00	0.00	.	.	
24<Monat	yes	18	0	0.00	0.00	0.00	0.00	0.00	0.00	0.00	0.00	.	.	
	no	18	0	0.00	0.00	0.00	0.00	0.00	0.00	0.00	0.00	.	.	
	Total	36	0	0.00	0.00	0.00	0.00	0.00	0.00	0.00	0.00	.	.	


Table 3.3: Number of IOP lowering eye drops	


Visit	Study eye	N	NMiss	Mean	STD	SEM	Min	Q1	Median	Q3	Max	Mean (95%LCL)	Mean (95%UCL)	
Pre surgery	yes	249	0	0.08	0.31	0.02	0.00	0.00	0.00	0.00	2.00	0.04	0.11	
	no	249	0	0.07	0.30	0.02	0.00	0.00	0.00	0.00	2.00	0.03	0.11	
	Total	498	0	0.07	0.31	0.01	0.00	0.00	0.00	0.00	2.00	0.05	0.10	
Post OP	yes	225	0	0.13	0.46	0.03	0.00	0.00	0.00	0.00	3.00	0.07	0.19	
	no	1	0	0.00	.	.	0.00	0.00	0.00	0.00	0.00	.	.	
	Total	226	0	0.13	0.46	0.03	0.00	0.00	0.00	0.00	3.00	0.07	0.19	
Discharge	yes	244	0	0.14	0.48	0.03	0.00	0.00	0.00	0.00	3.00	0.08	0.20	
	no	232	0	0.07	0.30	0.02	0.00	0.00	0.00	0.00	2.00	0.03	0.11	
	Total	476	0	0.11	0.40	0.02	0.00	0.00	0.00	0.00	3.00	0.07	0.14	
Entl<Monat<3	yes	180	0	0.10	0.38	0.03	0.00	0.00	0.00	0.00	3.00	0.04	0.16	
	no	101	0	0.05	0.22	0.02	0.00	0.00	0.00	0.00	1.00	0.01	0.09	
	Total	281	0	0.08	0.33	0.02	0.00	0.00	0.00	0.00	3.00	0.04	0.12	
3<=Monat<=6	yes	95	0	0.15	0.55	0.06	0.00	0.00	0.00	0.00	4.00	0.04	0.26	
	no	67	0	0.18	0.63	0.08	0.00	0.00	0.00	0.00	4.00	0.03	0.33	
	Total	162	0	0.16	0.58	0.05	0.00	0.00	0.00	0.00	4.00	0.07	0.25	
6<Monat<=12	yes	98	0	0.24	0.77	0.08	0.00	0.00	0.00	0.00	4.00	0.09	0.40	
	no	84	0	0.26	0.78	0.08	0.00	0.00	0.00	0.00	4.00	0.09	0.43	
	Total	182	0	0.25	0.77	0.06	0.00	0.00	0.00	0.00	4.00	0.14	0.37	
12<Monat<=24	yes	51	0	0.06	0.31	0.04	0.00	0.00	0.00	0.00	2.00	-0.03	0.15	
	no	50	0	0.06	0.31	0.04	0.00	0.00	0.00	0.00	2.00	-0.03	0.15	
	Total	101	0	0.06	0.31	0.03	0.00	0.00	0.00	0.00	2.00	-0.00	0.12	
24<Monat	yes	18	0	0.00	0.00	0.00	0.00	0.00	0.00	0.00	0.00	.	.	
	no	18	0	0.00	0.00	0.00	0.00	0.00	0.00	0.00	0.00	.	.	
	Total	36	0	0.00	0.00	0.00	0.00	0.00	0.00	0.00	0.00	.	.	
