# Supplementary material for: Influence of pars plana vitrectomy for macular surgery on the medium term intraocular pressure
Source: PLoS One. 2020 Oct 23;15(10):e0241005. doi: 10.1371/journal.pone.0241005 (PMC7584206; doi:10.1371/journal.pone.0241005)
Supplement: S2 Data — (RTF) [file pone.0241005.s002.rtf]

Primary analysis: 6<month<=12	
Complete cases	
Table 1.1: Complete cases - descriptiv statistics	

Study eye	N	Mean	STD	SEM	Min	Q1	Median	Q3	Max	Mean (95%LCL)	Mean (95%UCL)	
yes	84	-0.66	2.78	0.30	-8.00	-2.00	-0.42	1.00	6.00	-1.26	-0.06	
no	84	-0.17	2.98	0.33	-7.00	-2.00	0.00	2.00	7.00	-0.81	0.48	
Total	168	-0.41	2.89	0.22	-8.00	-2.00	0.00	1.75	7.00	-0.85	0.03	

Table 1.2: Complete cases - dependend t-test	

Variable	N	NMiss	Mean	SEM	STD	MIN	Q1	Median	Q3	MAX	t-Test: t-Value	t-Test: DF	t-Test: p-Value	Difference: LCL	Difference: UCL	
diff_auge	84	0	0.49	0.29	2.63	-7.00	-1.00	0.84	2.00	8.50	1.72	83	0.0893	-0.08	1.07	

Parameters: alpha=5%, H0=0	
Sensitivitiy analysis - LOCF	
Table 1.3: LOCF-Data - descriptiv statistics	

Study eye	N	Mean	STD	SEM	Min	Q1	Median	Q3	Max	Mean (95%LCL)	Mean (95%UCL)	
yes	249	-0.53	3.23	0.20	-9.00	-2.00	-1.00	1.00	15.00	-0.94	-0.13	
no	249	-0.35	2.67	0.17	-9.75	-1.50	0.00	1.00	8.00	-0.68	-0.02	
Total	498	-0.44	2.96	0.13	-9.75	-2.00	0.00	1.00	15.00	-0.70	-0.18	

Table 1.4: LOCF-Data - dependend t-test	

Variable	N	NMiss	Mean	SEM	STD	MIN	Q1	Median	Q3	MAX	t-Test: t-Value	t-Test: DF	t-Test: p-Value	Difference: LCL	Difference: UCL	
diff_auge	249	0	0.18	0.20	3.15	-16.00	-1.50	0.00	2.00	10.00	0.92	248	0.3559	-0.21	0.58	

Parameters: alpha=5%, H0=0	
Secondary analysis: 3<month<=6	
Complete cases	
Table 2.1: Complete cases - descriptiv statistics	

Study eye	N	Mean	STD	SEM	Min	Q1	Median	Q3	Max	Mean (95%LCL)	Mean (95%UCL)	
yes	65	-0.53	3.68	0.46	-7.00	-3.00	-0.50	1.00	15.00	-1.44	0.38	
no	65	-0.42	3.34	0.41	-10.00	-2.00	0.00	2.00	8.00	-1.25	0.41	
Total	130	-0.48	3.50	0.31	-10.00	-3.00	0.00	2.00	15.00	-1.08	0.13	

Table 2.2: Complete cases - dependend t-test	

Variable	N	NMiss	Mean	SEM	STD	MIN	Q1	Median	Q3	MAX	t-Test: t-Value	t-Test: DF	t-Test: p-Value	Difference: LCL	Difference: UCL	
diff_auge	65	0	0.11	0.45	3.61	-16.00	-1.00	0.00	2.00	9.00	0.24	64	0.8108	-0.79	1.00	

Parameters: alpha=5%, H0=0	
Sensitivitiy analysis - LOCF	
Table 2.3: LOCF-Data - descriptiv statistics	

Study eye	N	Mean	STD	SEM	Min	Q1	Median	Q3	Max	Mean (95%LCL)	Mean (95%UCL)	
yes	249	-0.28	3.23	0.20	-9.00	-2.00	-0.50	1.50	15.00	-0.68	0.12	
no	249	-0.35	2.49	0.16	-10.00	-1.50	0.00	1.00	8.00	-0.66	-0.04	
Total	498	-0.32	2.88	0.13	-10.00	-2.00	0.00	1.00	15.00	-0.57	-0.06	

Table 2.4: LOCF-Data - dependend t-test	

Variable	N	NMiss	Mean	SEM	STD	MIN	Q1	Median	Q3	MAX	t-Test: t-Value	t-Test: DF	t-Test: p-Value	Difference: LCL	Difference: UCL	
diff_auge	249	0	-0.07	0.20	3.10	-16.00	-2.00	0.00	2.00	9.00	-0.36	248	0.7195	-0.46	0.32	

Parameters: alpha=5%, H0=0	
Secondary analysis: 12<month<=24	
Complete cases	
Table 3.1: Complete cases - descriptiv statistics	

Study eye	N	Mean	STD	SEM	Min	Q1	Median	Q3	Max	Mean (95%LCL)	Mean (95%UCL)	
yes	50	-1.21	3.13	0.44	-7.00	-3.00	-1.50	0.50	10.00	-2.09	-0.32	
no	50	-1.16	2.96	0.42	-11.00	-2.00	-1.00	0.00	7.00	-2.01	-0.32	
Total	100	-1.18	3.03	0.30	-11.00	-2.75	-1.17	0.00	10.00	-1.79	-0.58	

Table 3.2: Complete cases - dependend t-test	

Variable	N	NMiss	Mean	SEM	STD	MIN	Q1	Median	Q3	MAX	t-Test: t-Value	t-Test: DF	t-Test: p-Value	Difference: LCL	Difference: UCL	
diff_auge	50	0	0.04	0.45	3.16	-9.00	-2.00	0.00	2.00	11.00	0.10	49	0.9231	-0.86	0.94	

Parameters: alpha=5%, H0=0	
Sensitivitiy analysis - LOCF	
Table 3.3: LOCF-Data - descriptiv statistics	

Study eye	N	Mean	STD	SEM	Min	Q1	Median	Q3	Max	Mean (95%LCL)	Mean (95%UCL)	
yes	249	-0.63	3.26	0.21	-9.00	-2.50	-1.00	1.00	15.00	-1.04	-0.22	
no	249	-0.42	2.69	0.17	-11.00	-2.00	0.00	1.00	8.00	-0.76	-0.08	
Total	498	-0.52	2.99	0.13	-11.00	-2.00	0.00	1.00	15.00	-0.79	-0.26	

Table 3.4: LOCF-Data - dependend t-test	

Variable	N	NMiss	Mean	SEM	STD	MIN	Q1	Median	Q3	MAX	t-Test: t-Value	t-Test: DF	t-Test: p-Value	Difference: LCL	Difference: UCL	
diff_auge	249	0	0.21	0.21	3.25	-16.00	-1.25	0.00	2.00	11.00	1.01	248	0.3145	-0.20	0.61	

Parameters: alpha=5%, H0=0	
Secondary analysis (additional influencing variables): ANCOVA (difference 6<month<=12 -pre surgery)	
	
Table 4.1: Study eye	

Class Level Information			
Class	Levels	Values	
Lense state	2	phak pseudophak	
Vitrectomy_	2	20G 23G	
Endotamponade_	4	C2F6 Air SF6 k	
Sex	2	male female	


Type 3 Tests of Fixed Effects					
Effect	Num DF	Den DF	F Value	Pr > F	
Lense state	1	87	0.28	0.5999	
Vitrectomy_	1	87	4.19	0.0436	
Endotamponade_	3	87	0.80	0.4982	
Sex	1	87	0.67	0.4136	
IOP_Mean_bl	1	87	20.34	<.0001	
Age_op	1	87	0.02	0.8773	
Sph_q	1	87	0.00	0.9768	
Anz_IOP-loweringAT_	1	87	0.06	0.8148	

Table 4.2: Both eyes	

Class Level Information			
Class	Levels	Values	
Study eye	2	yes no	
Lense state	2	phak pseudophak	
Sex	2	male female	


Type 3 Tests of Fixed Effects					
Effect	Num DF	Den DF	F Value	Pr > F	
Study eye	1	174	1.68	0.1964	
Lense state	1	174	0.17	0.6823	
Sex	1	174	0.02	0.8774	
IOP_Mean_bl	1	174	83.43	<.0001	
Age_op	1	174	0.09	0.7605	
Sph_q	1	174	0.09	0.7620	
Anz_IOP-loweringAT_	1	174	0.66	0.4167	

Sensitivitiy analysis - LOCF	
Table 5.1: Study eye	

Class Level Information			
Class	Levels	Values	
Lense state	2	phak pseudophak	
Vitrectomy_	2	20G 23G	
Endotamponade_	4	C2F6 Air SF6 k	
Sex	2	male female	


Type 3 Tests of Fixed Effects					
Effect	Num DF	Den DF	F Value	Pr > F	
Lense state	1	238	0.07	0.7877	
Vitrectomy_	1	238	2.62	0.1072	
Endotamponade_	3	238	2.18	0.0907	
Sex	1	238	2.10	0.1487	
IOP_Mean_bl	1	238	42.03	<.0001	
Age_op	1	238	0.35	0.5519	
Sph_q	1	238	0.07	0.7957	
Anz_IOP-loweringAT_	1	238	0.62	0.4307	

Table 5.2: Both eyes	

Class Level Information			
Class	Levels	Values	
Study eye	2	yes no	
Lense state	2	phak pseudophak	
Sex	2	male female	


Type 3 Tests of Fixed Effects					
Effect	Num DF	Den DF	F Value	Pr > F	
Study eye	1	490	0.16	0.6866	
Lense state	1	490	1.21	0.2717	
Sex	1	490	1.74	0.1876	
IOP_Mean_bl	1	490	133.50	<.0001	
Age_op	1	490	0.65	0.4222	
Sph_q	1	490	0.18	0.6723	
Anz_IOP-loweringAT_	1	490	0.93	0.3366	

Correlation: Prä-Op vs. 6<Monat<=12	

Table 6.1-1: Descriptive statistics	

Study eye	Variable	N	Mean	STD	Median	Minimum	Maximum	
yes	IOP_Mean	98	14.91	2.80	14.50	10.00	22.00	
yes	IOP_Mean_bl	98	15.66	2.72	16.00	10.00	24.00	
no	IOP_Mean	84	15.25	2.49	15.25	10.00	20.00	
no	IOP_Mean_bl	84	15.42	3.23	15.50	6.00	23.00	

Table 6.1-2: Correlation coefficient (Pearson and Spearman)	

	Data	Fisher's z-Transformation						
Study eye?	Correlation coefficient	N	Rho	Rho (Estimated))	LCL	UCL	p-value: H0:rho=0	
yes	Pearson (parametric)	98	0.5003	0.4984	0.3328	0.6341	<.0001	
	Spearman (non-parametric)	98	0.4792	0.4772	0.3080	0.6172	<.0001	
no	Pearson (parametric)	84	0.4796	0.4774	0.2929	0.6275	<.0001	
	Spearman (non-parametric)	84	0.4537	0.4515	0.2626	0.6072	<.0001	

Graph 6.2-1: Scatterplot	


	
Table 7.1: Comparison of the IOP-difference (fellow eye minus study-eye) with dependent t test	
Prä OP	

Variable	N	NMiss	Mean	SEM	STD	MIN	Q1	Median	Q3	MAX	t-Test: t-Value	t-Test: DF	t-Test: p-Value	Difference: LCL	Difference: UCL	
diff	249	0	-0.18	0.13	2.05	-8.00	-1.00	0.00	1.00	7.00	-1.36	248	0.1746	-0.43	0.08	

Parameters: alpha=5%, H0=0	
Discharge<Monat<=3	

Variable	N	NMiss	Mean	SEM	STD	MIN	Q1	Median	Q3	MAX	t-Test: t-Value	t-Test: DF	t-Test: p-Value	Difference: LCL	Difference: UCL	
diff	104	0	-0.97	0.34	3.42	-13.00	-2.25	0.00	1.00	6.00	-2.89	103	0.0047	-1.64	-0.31	

Parameters: alpha=5%, H0=0	
3<Monat<=6	

Variable	N	NMiss	Mean	SEM	STD	MIN	Q1	Median	Q3	MAX	t-Test: t-Value	t-Test: DF	t-Test: p-Value	Difference: LCL	Difference: UCL	
diff	65	0	-0.31	0.38	3.06	-15.00	-1.00	0.00	1.50	5.00	-0.81	64	0.4200	-1.07	0.45	

Parameters: alpha=5%, H0=0	
6<Monat<=12	

Variable	N	NMiss	Mean	SEM	STD	MIN	Q1	Median	Q3	MAX	t-Test: t-Value	t-Test: DF	t-Test: p-Value	Difference: LCL	Difference: UCL	
diff	84	0	0.20	0.20	1.86	-4.50	-1.00	0.00	1.00	4.00	0.97	83	0.3348	-0.21	0.60	

Parameters: alpha=5%, H0=0	
12<Monat<=24	

Variable	N	NMiss	Mean	SEM	STD	MIN	Q1	Median	Q3	MAX	t-Test: t-Value	t-Test: DF	t-Test: p-Value	Difference: LCL	Difference: UCL	
diff	50	0	0.08	0.37	2.58	-7.00	-1.00	0.00	1.00	9.50	0.23	49	0.8203	-0.65	0.82	

Parameters: alpha=5%, H0=0	
24<Monat	

Variable	N	NMiss	Mean	SEM	STD	MIN	Q1	Median	Q3	MAX	t-Test: t-Value	t-Test: DF	t-Test: p-Value	Difference: LCL	Difference: UCL	
diff	18	0	0.22	0.37	1.56	-3.00	0.00	0.00	1.00	4.00	0.60	17	0.5547	-0.56	1.00	

Parameters: alpha=5%, H0=0	
