# Supplementary material for: Influence of pars plana vitrectomy for macular surgery on the medium term intraocular pressure
Source: PLoS One. 2020 Oct 23;15(10):e0241005. doi: 10.1371/journal.pone.0241005 (PMC7584206; doi:10.1371/journal.pone.0241005)
Supplement: S3 Data — (RTF) [file pone.0241005.s003.rtf]

Baseline-Charakteristics	
Table 1.1: Age at OP + Sex	

Variable	Sex	N	NMiss	Mean	SEM	STD	MIN	Q1	Median	Q3	MAX	Method	t-Test:
t-Value	t-Test:
DF	t-Test:
p-Value	Difference:
Mean	Difference:
LCL	Difference:
UCL	
Age_op	female	137	0	70.07	0.56	6.56	54.05	65.66	69.94	73.56	100.70	Pooled	0.08	247	0.9343	0.07	-1.51	1.64	
	male	112	0	70.01	0.56	5.91	44.59	67.49	70.13	73.62	83.97		.	.	.	.	.	.	

Parameters: alpha=5%, H0=0	


N	NMiss	Mean	STD	SEM	Min	Q1	Median	Q3	Max	Mean (95%LCL)	Mean (95%UCL)	
249	0	70.04	6.26	0.40	44.59	66.84	70.05	73.62	100.70	69.26	70.82	

Table 1.2: Systemic steroids	


	Systemic steroids		
Sex	inhalativ (i)	intranasal (i.n.)	oral (p.o.)	none (k)	Total	
female	5 (3.6%)	1 (0.7%)	4 (2.9%)	127 (92.7%)	137 (55%)	
male	2 (1.8%)	0 (0%)	1 (0.9%)	109 (97.3%)	112 (45%)	
Total	7 (2.8%)	1 (0.4%)	5 (2%)	236 (94.8%)	249 (100%)	


Table 1.3: Study eye	


	Study eye		
Sex	left	right	Total	
female	73 (53.3%)	64 (46.7%)	137 (55%)	
male	51 (45.5%)	61 (54.5%)	112 (45%)	
Total	124 (49.8%)	125 (50.2%)	249 (100%)	


Baseline characteristics	
Table 2.1: IOP - Baseline - Descriptiv statistics + Dependent 2 sided t test	


Study eye	N	NMiss	Mean	STD	SEM	Min	Q1	Median	Q3	Max	Mean (95%LCL)	Mean (95%UCL)	
yes	249	0	15.67	2.81	0.18	9.00	14.00	16.00	17.00	24.00	15.32	16.02	
no	249	0	15.49	2.90	0.18	6.00	14.00	15.00	18.00	23.00	15.13	15.86	
Total	498	0	15.58	2.85	0.13	6.00	14.00	16.00	18.00	24.00	15.33	15.83	


Variable	N	NMiss	Mean	SEM	STD	MIN	Q1	Median	Q3	MAX	t-Test:
t-Value	t-Test:
DF	t-Test:
p-Value	Difference:
LCL	Difference:
UCL	
diff	249	0	-0.18	0.13	2.05	-8.00	-1.00	0.00	1.00	7.00	-1.36	248	0.1746	-0.43	0.08	

Parameters: alpha=5%, H0=0	
Table 2.2: Spherical equivalent - Descriptiv statistics + Dependent 2 sided t test	


Study eye	N	NMiss	Mean	STD	SEM	Min	Q1	Median	Q3	Max	Mean (95%LCL)	Mean (95%UCL)	
yes	249	0	0.35	2.60	0.16	-15.00	-0.50	0.75	2.00	7.00	0.03	0.67	
no	249	0	0.45	2.63	0.17	-12.00	-0.25	1.00	2.00	7.50	0.12	0.78	
Total	498	0	0.40	2.61	0.12	-15.00	-0.25	0.75	2.00	7.50	0.17	0.63	


Variable	N	NMiss	Mean	SEM	STD	MIN	Q1	Median	Q3	MAX	t-Test:
t-Value	t-Test:
DF	t-Test:
p-Value	Difference:
LCL	Difference:
UCL	
diff	249	0	0.10	0.08	1.19	-4.50	-0.50	0.00	0.50	8.50	1.37	248	0.1732	-0.05	0.25	

Parameters: alpha=5%, H0=0	

Table 2.3: Diagnosis	


	Diagnosis		
Study eye	Epiretinale Gliose (EG)	Makulaforamen (MF)	Vitroretinales Traktionssyndrom (VRT)	MF+EG	VMT	none (k)	Total	
yes	140 (56.2%)	100 (40.2%)	3 (1.2%)	4 (1.6%)	2 (0.8%)	0 (0%)	249	
no	0 (0%)	0 (0%)	0 (0%)	0 (0%)	0 (0%)	249 (100%)	249	
Total	140	100	3	4	2	249	498	

Table 2.4: Vitrectomy	


	Vitrectomy		
Study eye	20G	23G	k	Total	
yes	202 (81.1%)	47 (18.9%)	0 (0%)	249	
no	0 (0%)	0 (0%)	249 (100%)	249	
Total	202	47	249	498	

Table 2.5: Endotamponade	


	Endotamponade		
Study eye	C2F6	Luft	SF6	k	Total	
yes	39 (15.7%)	19 (7.6%)	73 (29.3%)	118 (47.4%)	249	
no	0 (0%)	0 (0%)	0 (0%)	249 (100%)	249	
Total	39	19	73	367	498	

Table 2.6: Peeing dye	


	Peeing dye		
Study eye	Brilliant Blau (BB)	Brilliant Peel (BP)	Dual Blue (DB)	Methylenblau (MB)	Indocyaningrün (ICG)	Trypanblau (TB)	ILM-Blue	none (k)	Total	
yes	27 (10.8%)	103 (41.4%)	16 (6.4%)	1 (0.4%)	23 (9.2%)	1 (0.4%)	22 (8.8%)	56 (22.5%)	249	
no	0 (0%)	0 (0%)	0 (0%)	0 (0%)	0 (0%)	0 (0%)	0 (0%)	249 (100%)	249	
Total	27	103	16	1	23	1	22	305	498	

Table 2.7: Lense state	


	Lense state		
Study eye	phak	pseudophak	Total	
yes	231 (92.8%)	18 (7.2%)	249	
no	232 (93.2%)	17 (6.8%)	249	
Total	463	35	498	

Table 3.1: IOP (Intraocular pressure)	


Visit	Study eye	N	NMiss	Mean	STD	SEM	Min	Q1	Median	Q3	Max	Mean (95%LCL)	Mean (95%UCL)	
Prä OP	yes	249	0	15.67	2.81	0.18	9.00	14.00	16.00	17.00	24.00	15.32	16.02	
	no	249	0	15.49	2.90	0.18	6.00	14.00	15.00	18.00	23.00	15.13	15.86	
	Total	498	0	15.58	2.85	0.13	6.00	14.00	16.00	18.00	24.00	15.33	15.83	
Post OP	yes	225	0	17.60	5.42	0.36	2.00	14.00	17.00	20.00	39.00	16.89	18.32	
	no	1	0	13.00	.	.	13.00	13.00	13.00	13.00	13.00	.	.	
	Total	226	0	17.58	5.41	0.36	2.00	14.00	17.00	20.00	39.00	16.87	18.29	
Discharge	yes	244	0	15.88	4.56	0.29	4.00	13.00	16.00	18.00	33.00	15.30	16.45	
	no	232	0	14.94	3.05	0.20	6.00	13.00	15.00	17.00	23.00	14.55	15.34	
	Total	476	0	15.42	3.92	0.18	4.00	13.00	15.00	18.00	33.00	15.07	15.78	
Entl<Monat<=3	yes	184	0	15.68	3.70	0.27	6.00	13.50	15.75	18.00	28.00	15.14	16.22	
	no	104	0	14.46	2.76	0.27	9.00	12.00	14.25	16.00	24.00	13.93	15.00	
	Total	288	0	15.24	3.44	0.20	6.00	13.00	15.00	17.00	28.00	14.84	15.64	
3<Monat<=6	yes	91	0	14.68	3.63	0.38	5.00	13.00	14.00	16.00	30.00	13.92	15.43	
	no	65	0	14.67	3.04	0.38	8.00	12.50	14.50	16.00	23.00	13.92	15.42	
	Total	156	0	14.67	3.38	0.27	5.00	13.00	14.00	16.00	30.00	14.14	15.21	
6<Monat<=12	yes	98	0	14.91	2.80	0.28	10.00	13.00	14.50	17.00	22.00	14.35	15.48	
	no	84	0	15.25	2.49	0.27	10.00	14.00	15.25	17.00	20.00	14.71	15.79	
	Total	182	0	15.07	2.66	0.20	10.00	13.00	15.00	17.00	22.00	14.68	15.46	
12<Monat<=24	yes	51	0	14.72	3.29	0.46	7.50	12.00	15.00	17.67	24.00	13.79	15.64	
	no	50	0	14.74	2.73	0.39	6.00	13.00	15.00	16.00	20.50	13.96	15.51	
	Total	101	0	14.73	3.01	0.30	6.00	13.00	15.00	17.00	24.00	14.13	15.32	
24<Monat	yes	18	0	14.93	2.49	0.59	10.67	13.00	15.00	16.00	20.00	13.69	16.16	
	no	18	0	15.15	2.40	0.57	10.67	14.00	15.00	17.00	21.00	13.96	16.34	
	Total	36	0	15.04	2.41	0.40	10.67	13.50	15.00	16.50	21.00	14.22	15.85	


Table 3.2: Number of steroid eye drops (only Study eye)	


	Steroid eye drops		
Visit	Study eye	no	yes	Total	
Prä OP	yes	249 (100%)	0	249	
	no	249 (100%)	0	249	
	Total	498	0	498	
Post OP	yes	1 (0.4%)	224 (99.6%)	225	
	no	1 (100%)	0 (0%)	1	
	Total	2	224	226	
Discharge	yes	2 (0.8%)	242 (99.2%)	244	
	no	232 (100%)	0 (0%)	232	
	Total	234	242	476	
Entl<Monat<=3	yes	155 (84.2%)	29 (15.8%)	184	
	no	104 (100%)	0 (0%)	104	
	Total	259	29	288	
3<Monat<=6	yes	90 (98.9%)	1 (1.1%)	91	
	no	65 (100%)	0 (0%)	65	
	Total	155	1	156	
6<Monat<=12	yes	98 (100%)	0	98	
	no	84 (100%)	0	84	
	Total	182	0	182	
12<Monat<=24	yes	51 (100%)	0	51	
	no	50 (100%)	0	50	
	Total	101	0	101	
24<Monat	yes	18 (100%)	0	18	
	no	18 (100%)	0	18	
	Total	36	0	36	


Number of IOP lowering eye drops	
Table 3.3-1: Number of IOP lowering eye drops 	
	


	Number of IOP lowering eye drops		
Visit	Study eye	0	1	1.5	2	3	Total	
Prä OP	yes	233 (93.6%)	13 (5.2%)	0	3 (1.2%)	0	249	
	no	234 (94%)	12 (4.8%)	0	3 (1.2%)	0	249	
	Total	467	25	0	6	0	498	
Post OP	yes	206 (91.6%)	10 (4.4%)	0	8 (3.6%)	1 (0.4%)	225	
	no	1 (100%)	0 (0%)	0	0 (0%)	0 (0%)	1	
	Total	207	10	0	8	1	226	
Discharge	yes	220 (90.2%)	15 (6.1%)	0	7 (2.9%)	2 (0.8%)	244	
	no	219 (94.4%)	10 (4.3%)	0	3 (1.3%)	0 (0%)	232	
	Total	439	25	0	10	2	476	
Entl<Monat<=3	yes	170 (92.4%)	11 (6%)	1 (0.5%)	2 (1.1%)	0	184	
	no	98 (94.2%)	6 (5.8%)	0 (0%)	0 (0%)	0	104	
	Total	268	17	1	2	0	288	
3<Monat<=6	yes	82 (90.1%)	7 (7.7%)	0	2 (2.2%)	0	91	
	no	58 (89.2%)	5 (7.7%)	0	2 (3.1%)	0	65	
	Total	140	12	0	4	0	156	
6<Monat<=12	yes	86 (87.8%)	8 (8.2%)	1 (1%)	3 (3.1%)	0	98	
	no	72 (85.7%)	8 (9.5%)	1 (1.2%)	3 (3.6%)	0	84	
	Total	158	16	2	6	0	182	
12<Monat<=24	yes	49 (96.1%)	2 (3.9%)	0	0	0	51	
	no	48 (96%)	2 (4%)	0	0	0	50	
	Total	97	4	0	0	0	101	
24<Monat	yes	18 (100%)	0	0	0	0	18	
	no	18 (100%)	0	0	0	0	18	
	Total	36	0	0	0	0	36	


Table 3.3-2: Number of IOP lowering eye drops	


Visit	Study eye	N	NMiss	Mean	STD	SEM	Min	Q1	Median	Q3	Max	Mean (95%LCL)	Mean (95%UCL)	
Prä OP	yes	249	0	0.08	0.31	0.02	0.00	0.00	0.00	0.00	2.00	0.04	0.11	
	no	249	0	0.07	0.30	0.02	0.00	0.00	0.00	0.00	2.00	0.03	0.11	
	Total	498	0	0.07	0.31	0.01	0.00	0.00	0.00	0.00	2.00	0.05	0.10	
Post OP	yes	225	0	0.13	0.46	0.03	0.00	0.00	0.00	0.00	3.00	0.07	0.19	
	no	1	0	0.00	.	.	0.00	0.00	0.00	0.00	0.00	.	.	
	Total	226	0	0.13	0.46	0.03	0.00	0.00	0.00	0.00	3.00	0.07	0.19	
Discharge	yes	244	0	0.14	0.48	0.03	0.00	0.00	0.00	0.00	3.00	0.08	0.20	
	no	232	0	0.07	0.30	0.02	0.00	0.00	0.00	0.00	2.00	0.03	0.11	
	Total	476	0	0.11	0.40	0.02	0.00	0.00	0.00	0.00	3.00	0.07	0.14	
Entl<Monat<=3	yes	184	0	0.09	0.33	0.02	0.00	0.00	0.00	0.00	2.00	0.04	0.14	
	no	104	0	0.06	0.23	0.02	0.00	0.00	0.00	0.00	1.00	0.01	0.10	
	Total	288	0	0.08	0.30	0.02	0.00	0.00	0.00	0.00	2.00	0.04	0.11	
3<Monat<=6	yes	91	0	0.12	0.39	0.04	0.00	0.00	0.00	0.00	2.00	0.04	0.20	
	no	65	0	0.14	0.43	0.05	0.00	0.00	0.00	0.00	2.00	0.03	0.24	
	Total	156	0	0.13	0.41	0.03	0.00	0.00	0.00	0.00	2.00	0.06	0.19	
6<Monat<=12	yes	98	0	0.16	0.45	0.05	0.00	0.00	0.00	0.00	2.00	0.07	0.25	
	no	84	0	0.18	0.48	0.05	0.00	0.00	0.00	0.00	2.00	0.08	0.29	
	Total	182	0	0.17	0.47	0.03	0.00	0.00	0.00	0.00	2.00	0.10	0.24	
12<Monat<=24	yes	51	0	0.04	0.20	0.03	0.00	0.00	0.00	0.00	1.00	-0.02	0.09	
	no	50	0	0.04	0.20	0.03	0.00	0.00	0.00	0.00	1.00	-0.02	0.10	
	Total	101	0	0.04	0.20	0.02	0.00	0.00	0.00	0.00	1.00	0.00	0.08	
24<Monat	yes	18	0	0.00	0.00	0.00	0.00	0.00	0.00	0.00	0.00	.	.	
	no	18	0	0.00	0.00	0.00	0.00	0.00	0.00	0.00	0.00	.	.	
	Total	36	0	0.00	0.00	0.00	0.00	0.00	0.00	0.00	0.00	.	.	
